# Effect of the rs12249854 Polymorphism in the *HECTD2* Gene and Selected Clinical Variables on the Response to Intradetrusor Injection of Botulinum Neurotoxin Type a in Female Patients with Overactive Bladder: A Single-Center Prospective Study

**DOI:** 10.3390/ijms27219571

**Published:** 2026-10-26

**Authors:** Sylwester Michał Ciećwież, Klaudyna Lewandowska, Agnieszka Boroń, Dariusz Kotlęga, Jacek Kociszewski, Andrzej Ciechanowicz

**Affiliations:** 1 Department of Perinatology, Obstetrics and Gynecology, Pomeranian Medical University, 70-204 Szczecin, Poland; 2 Department of Clinical and Molecular Biochemistry, Pomeranian Medical University, 70-204 Szczecin, Poland; klaudyna.lewandowskal@pum.edu.pl (K.L.); agnieszka.boron@pum.edu.pl (A.B.); andrzej.ciechanowicz@pum.edu.pl (A.C.); 3 Department of Pharmacology and Toxicology, University of Zielona Góra, 65-417 Zielona Góra, Poland; d.kotlega@inz.uz.zgora.pl; 4 Department of Gynecology, Evangelisches Krankenhaus Hagen, 58135 Hagen, Germany; kociszewski64@gmail.com

**Keywords:** idiopathic overactive bladder, neurogenic overactive bladder, botulinum toxin, treatment response, urodynamic parameters, patient-reported measures, ICIQ-OAB, ICIQ—LUTS-QoL, pharmacogenetics, genetic predictors

## Abstract

The *HECTD2*:rs12249854 polymorphism may be associated with a weaker response to an intradetrusor injection of botulinum neurotoxin type A (BoNT/A) in patients with overactive bladder (OAB). This prospective study analyzed the link between the *HECTD2*:rs12249854 polymorphism and the response to BoNT/A treatment in 156 OAB patients, based on changes in urodynamic parameters and ICIQ-OAB and ICIQ-LUTS-QoL scores six months after the injection. BoNT/A treatment contributed to improvements in all parameters except the average flow rate. Wild-type homozygotes (TT, *n* = 137) differed from TA heterozygotes (*n* = 19) solely in terms of pretreatment ICIQ-OAB Part A and ICIQ-LUTS-QoL Part A scores, both of which were higher in the heterozygotes. Thus, the *HECTD2*:rs12249854 polymorphism does not appear to be associated with the response to BoNT/A treatment. Patients with neurogenic OAB (*n* = 60) had a higher pretreatment detrusor pressure at maximum flow than those with idiopathic OAB (*n* = 96), but this difference was no longer observed after BoNT/A treatment. Good responders to the BoNT/A injection (defined as at least 50% decrease in the ICIQ-LUTS-QoL Part A score, *n* = 50) had a higher body mass index and parity and a lower pretreatment maximum flow rate than poor responders (*n* = 106). Before formulating any definitive conclusions, the results of this exploratory study should be verified in an adequately powered multicenter cohort.

## 1. Introduction

According to the International Continence Society (ICS), overactive bladder (OAB) is a syndrome characterized by involuntary contractions of the bladder muscles, specifically the detrusor [1]. OAB is diagnosed based on the occurrence of four symptoms: urgency, i.e., a sudden and immediate need to pass urine; frequency, i.e., the need to urinate more often than usual, typically at least eight times per day; nocturia, i.e., waking up at night at least twice because of a strong urge to pass urine; and urge incontinence, i.e., involuntary leakage of urine [2,3]. In most cases, the root cause of detrusor overactivity is unknown, leading to a diagnosis of idiopathic OAB (iOAB) [4]. A smaller fraction of patients suffer from a typically more severe form of OAB, neurogenic OAB (nOAB), in which the detrusor dysfunction results from an underlying neurological problem, e.g., multiple sclerosis, spinal cord injury, stroke, etc. [5,6,7].

The global prevalence of OAB is estimated at 20% of the adult population, with a higher prevalence in women than in men [8,9]. The prevalence of OAB increases with age, with the occurrence peaking in sexagenarians [10]. Other risk factors for OAB include overweight and obesity, bladder outlet obstruction, diabetes mellitus, psychological stress, lower socioeconomic status, and genetic predisposition [8,9]. OAB has a detrimental effect on quality of life, contributing to social isolation, a sense of stigmatization, depression, and sleep disorders, while also negatively affecting sexual function [11]. Despite this, the condition appears to be severely undertreated, with less than 50% of affected patients seeking medical advice [12,13].

Intradetrusor injection of botulinum neurotoxin type A (BoNT/A) has emerged as a promising treatment for OAB [14,15,16]. While BoNT/A appears to be generally effective in controlling OAB symptoms, published data suggest that not all patients respond to the treatment equally well [17,18]. This has stimulated research on response markers and, given the established genetic predisposition to OAB, specifically on pharmacogenetic ones. Several genes that could theoretically be involved in the pathophysiology of overactive bladder, including the *SV2*, *SNAP25*, *ADRB3*, and *BDNF* genes, have been tested recently for their link with syndrome severity and/or resistance to BoNT/A treatment. However, the results of those studies, also conducted by our group, are still inconclusive, raising interest in alternative candidate genes and their polymorphisms [19,20,21,22,23].

Botulinum neurotoxins consist of an N-terminal zinc metalloprotease light chain (LC) disulfide-linked to a C-terminal heavy chain (HC) [24,25]. The HC binds to specific receptors on the presynaptic terminal and delivers the LC protease into the cell [26]. Loop 4 of the luminal glycosylated domain of a synaptic vesicle protein (SV2) has been identified as the protein receptor interface for BoNT/A [21]. After entering the nerve terminal cytosol, the LC of BoNT/A cleaves the synaptosomal-associated 25 kDa protein (SNAP-25) at its carboxyl-terminal peptide bond Gln197-Arg198, thus blocking neurotransmitter release and resulting in paralysis that lasts 2–6 months after a single exposure to the toxin [27,28,29]. One of the key factors contributing to the duration of paralysis is the relative lifetime of the LC of BoNT/A in the presynaptic terminal [28,30,31]. The LC of BoNT/A has been shown to persist by evading proteasomal degradation because of its association with a deubiquitinating enzyme, valosin-containing protein complex-interacting protein 135 kDa (VCIP135) [24]. In the absence of VCIP135, the light chain of BoNT/A is targeted for degradation by the homologous to E6-AP carboxy-terminus protein (HECT) domain E3 ubiquitin protein ligase 2 [24].

Human HECT domain E3 ubiquitin protein ligase 2 is encoded by the *HECTD2* gene located on chromosome 10q23.32 [32]. The *HECTD2*:rs12249854 polymorphism is a T-to-A transversion located at NC_000010.11:g.91426505T>A according to the genome assembly GRCh38.p14 chromosome 10 [33]. The rs12249854 polymorphism is in complete linkage disequilibrium with the rs7081363 polymorphism located in the *HECTD2* promoter (-247G>A) and disrupts an Sp1 binding site [34]. The expression levels of *HECTD2* mRNA in the blood lymphocytes of *HECTD2*:rs12249854 heterozygotes (TA genotype) have been shown to be over 62 times higher than in wild-type allele homozygotes (TT genotype) [34]. Therefore, the question arises whether the *HECTD2*:rs12249854 polymorphism might be associated with the response to a single injection of BoNT/A in female patients with OAB.

The aim of this prospective study was to analyze the link between the rs12249854 polymorphism in the *HECTD2* gene and the response to BoNT/A treatment in a cohort of women with iOAB and nOAB.

## 2. Results

Our series included 137 TT homozygotes (87.3%), 19 TA heterozygotes (12.1%), and 1 AA homozygote (0.6%). The frequency of the minor *HECTD2*:rs12249854 allele (A allele) was 6.7%. Patients with iOAB did not differ from those with nOAB in the frequency distributions of *HECTD2* genotypes (84.5% TT, 14.4% TA and 1.1% AA versus 91.7% TT, 8.3% TA and 0.0% AA, *p* = 0.372, for iOAB and nOAB, respectively) or alleles (91.8% T and 8.2% A versus 95.6% T and 4.2% A, *p* = 0.159, for iOAB and nOAB, respectively). The *HECTD2*:rs12249854 (GRCh38.p14: NC_000010.11:g.91426505T>A) genotype distributions in the iOAB group, nOAB group, and the entire series were consistent with Hardy–Weinberg equilibrium (*p* = 0.648, *p* = 0.736 and *p* = 0.703, respectively).

To analyze the association between the *HECTD2* polymorphism and the response to BoNT/A treatment, individuals with iOAB and nOAB were pooled into a single group. After excluding the single AA homozygote, the results for wild-type allele homozygotes (TT genotype) were compared with those for polymorphic heterozygotes (TA genotype).

TT homozygotes did not differ significantly from TA heterozygotes in terms of age, body mass index, or the number of pregnancies, deliveries, or cesarean sections (Table 1).

Except for pretreatment scores for ICIQ-OAB Part A and ICIQ-LUTS-QoL Part A, the TT homozygotes did not differ significantly from the TA heterozygotes in terms of any other patient-reported outcome measures determined before treatment and six months thereafter, or in terms of absolute and relative differences at six months. The scores for ICIQ-OAB Part A and ICIQ-LUTS-QoL Part A in the TA heterozygotes were significantly higher than in the wild-type allele *HECTD2* homozygotes (Table 2).

The TT homozygotes did not differ significantly from the TA heterozygotes in terms of any of the analyzed urodynamic parameters (Table 3). Also, no significant differences were found between the genotype groups in the frequency of complete bladder emptying before BoNT/A injection (61/137, 44.5% versus 9/19, 47.4%, *p* = 0.934), six months thereafter (54/137, 39.4% versus 9/19, 47.4%, *p* = 0.574), or both before treatment and at six months (28/137, 20.48% versus 6/19, 31.6%, *p* = 0.372).

After excluding the single AA homozygote, the study cohort consisted of 96 patients with iOAB and 60 women with nOAB. In the majority of nOAB patients (*n* = 46, 76.7%), multiple sclerosis was the underlying diagnosis. The other neurological diagnoses in the nOAB group included lumbar discopathy (*n* = 7, 11.7%), myelitis (*n* = 5, 8.3%), and Devic’s disease (*n* = 2, 3.3%).

Patients with iOAB did not differ significantly from those with nOAB in terms of age, body mass index, or the number of pregnancies, deliveries, or cesarean sections (Table 4), or in terms of the scores for ICIQ-OAB Part A, ICIQ-OAB Part B, ICIQ-LUTS-QoL Part A, and ICIQ-LUTS-QoL Part B before treatment and six months after BoNT/A injection (Table 5).

Except for pretreatment values of Pdet at Qmax, no significant between-group differences were found in the analyzed urodynamic parameters. The pretreatment Pdet at Qmax in patients with nOAB was significantly higher than in individuals with iOAB (Table 6). Patients with iOAB did not differ significantly from those with nOAB in the frequency of complete bladder emptying before BoNT/A injection (48/96, 50.0% versus 22/60, 36.7%, *p* = 0.103) or six months after treatment (42/96, 43.8% versus 21/60, 35.0%, *p* = 0.279). However, the proportion of patients with complete bladder emptying both before treatment and six months thereafter was significantly higher in the iOAB group than in the nOAB group (27/96, 28.1% versus 7/60, 11.7%, *p* = 0.017).

Repeated-measures ANOVA demonstrated that BoNT/A treatment contributed to statistically significant changes in all analyzed parameters except for Qave (Supplementary Tables S1 and S2).

The rs12249854 polymorphism in the *HECTD2* gene exerted significant main effects on the scores for Part A of the ICIQ-OAB questionnaire (F = 4.19, *p* = 0.042) and Part A of the ICIQ-LUTS-QoL questionnaire (F = 5.37, *p* = 0.022), but no significant interaction effects were found with either treatment or OAB type (Supplementary Table S1).

Good treatment response was defined as at least a 50% relative decrease in the score for ICIQ-LUTS-QoL Part A within six months of treatment. The only three parameters that distinguished good responders from patients who did not respond adequately to BoNT/A injection were significantly higher BMI and parity and significantly lower pretreatment values of Qmax (Table 7).

Good responders did not differ significantly from poor responders in the frequency of complete bladder emptying before BoNT/A injection (30/50, 60.0% versus 56/106, 52.8%, *p* = 0.401), six months thereafter (32/50, 64.0% versus 61/106, 57.5%, *p* = 0.443), or both before treatment and at six months (10/50, 20.0% versus 24/106, 22.6%, *p* = 0.709).

## 3. Discussion

In the present prospective observational study, the presence of the rs12249854 polymorphism in the *HECTD2* gene had no significant effect on the characteristics of patients with OAB, other than the pretreatment scores for two questionnaires, ICIQ-OAB Part A and ICIQ-LUTS-QoL Part A. While patients with nOAB had significantly higher pretreatment values of Pdet at Qmax than individuals with iOAB, the between-group difference in this parameter was no longer significant six months after BoNT/A treatment. Intradetrusor injection of BoNT/A contributed to a significant improvement in all analyzed patient-reported measures and all urodynamic parameters except Qave. The outcome of BoNT/A treatment was not significantly modulated by either the *HECTD2* genotype or OAB type. However, the study identified three other potential correlates of the treatment outcome: BMI, number of pregnancies, and baseline Qmax.

Patients with one polymorphic allele of the *HECTD2* gene (TA genotype) differed significantly from wild-type allele homozygotes (TT genotype) in terms of two self-reported measures: the pretreatment scores for ICIQ-OAB Part A and ICIQ-LUTS-QoL Part A questionnaires. While Part A of the ICIQ-OAB questionnaire measures the frequency and severity of four core OAB symptoms—frequency, nocturia, urgency, and urge incontinence—Part A of the ICIQ-LUTS-QoL determines the impact of those symptoms on overall quality of life, daily functioning, and well-being [35,36]. The fact that patients with one polymorphic allele presented with significantly higher pretreatment scores for both questionnaires implies that their subjectively determined condition before treatment was worse than in wild-type allele homozygotes. This observation, however, was not confirmed in the analysis of objective urodynamic parameters. This suggests that the significant effect of the *HECTD2* polymorphism on the two questionnaire scores was likely an incidental finding, especially since no published evidence supports a link between the function of this gene and OAB severity.

When planning this study, we hypothesized that the presence of the rs12249854 polymorphism in the *HECTD2* gene might be associated with a less favorable outcome of BoNT/A treatment in patients with OAB. This hypothesis was based on published evidence that the expression of *HECTD2* mRNA in *HECTD2*:rs12249854 heterozygotes (TA genotype) is considerably higher than in wild-type allele homozygotes (TT genotype) [34]. Hence, one would expect patients with the TA genotype to present with considerably higher levels of HECT domain E3 ubiquitin protein ligase 2, an enzyme responsible for the degradation of BoNT/A [24].

Although the rs12249854 polymorphism in the *HECTD2* gene did not exert a significant effect on the outcome of BoNT/A treatment in our series, the results of this study do not necessarily mean that such a relationship does not exist. Our sample included only one polymorphic homozygote (AA genotype), and this single patient was excluded from the analysis. Perhaps the presence of only one polymorphic allele in the remaining patients was not enough for the biological effects of the rs12249854 polymorphism on BoNT/A degradation to manifest fully. Also, the fact that the *HECTD2*:rs12249854 heterozygotes (women with the TA genotype) were underrepresented in our cohort (19 out of 156 patients) should be considered a serious flaw. Nonetheless, the frequency of the minor rs12249854 allele (A allele) in our series closely resembled that documented in Europeans (4.6–4.8%) [37]. Thus, unless an adequately powered dedicated multicenter study or pooled analysis of AA homozygotes versus wild-type allele *HECTD2* homozygotes and TA heterozygotes is conducted, the effect of the rs12249854 polymorphism, if any, may not be captured.

The identification of genetic markers for OAB appears to be challenging. Despite a large body of evidence suggesting their potential role in the pathogenesis of the disease, polymorphisms in two genes—*ADRB3* and *BDNF*—failed to be identified as standalone markers for OAB [19,20,21,22,23]. Moreover, extensive analyses of available human genomic databases excluded a role for polymorphisms in the genes encoding two critical membrane-bound proteins, SV2 and SNAP25, in the development of resistance to BoNT/A [19,20]. Aside from the aforementioned problem regarding the underrepresentation of polymorphic homozygotes, the complexity of the disease may be another issue. It cannot be excluded that the clinical phenotype of OAB is the outcome of an interaction between several genes. If this is the case, a more complex predictive model involving more than one genetic alteration and perhaps also some non-genetic variables should be tested for its accuracy instead of focusing on a single polymorphism in a given gene.

Six months after intradetrusor injection of BoNT/A, we documented significant improvements in all analyzed clinical and patient-reported outcome measures except Qave. Thus, consistent with previous studies, BoNT/A proved to be an effective treatment for OAB, improving both the clinical condition of the patients and their overall well-being [38,39,40,41,42]. The lack of improvement in Qave is not surprising given that this parameter represents the total volume of urine voided, divided by the urination time [43]. The injection of BoNT/A weakens detrusor contractility, which is reflected by a decrease in Qmax. To compensate for a lower peak pressure, the bladder emptying time may increase slightly. A pretreatment flow curve in OAB patients is typically narrow, with a high peak corresponding to elevated Qmax. After BoNT/A treatment, the curve drops in height due to the reduction in Qmax but spreads over a longer time horizon, assuming the total volume of voided urine remains the same. As a result, the area under the curve remains essentially the same. In this way, a mild drop in Qmax can be mathematically masked by a steady, prolonged flow, leaving the overall average unchanged.

In the present study, patients with nOAB presented with less favorable baseline values of Pdet at Qmax than individuals with iOAB. This finding was to be expected given that nOAB is known to have a worse prognosis than iOAB. In particular, patients with nOAB are at an increased risk of detrusor-sphincter dyssynergia with resultant urinary retention and a predisposition to severe urinary tract infections [44,45,46]. However, the severity of nOAB varies and is determined by the underlying neurological condition, with the least favorable outcomes reported in patients with spinal cord injury and a much better prognosis in those with cerebrovascular accidents [7,47,48]. Our group of nOAB patients included a considerable proportion (76.7%) of women with multiple sclerosis, a condition in which the likelihood of detrusor-sphincter dyssynergia is particularly high [49,50]. However, our sample also included patients with other neurological diagnoses, and given the heterogeneity of this subset, we did not consider the type of underlying disorder as an exploratory variable in the analysis. Thus, the higher pretreatment values of Pdet at Qmax documented in the nOAB group should be interpreted with caution. Nonetheless, our patients with nOAB and iOAB did not differ in terms of post-treatment values of Pdet at Qmax or the proportions of good responders, so individuals with both OAB types appeared to benefit equally from BoNT/A treatment.

We also searched for potential correlates of a good therapeutic response to BoNT/A treatment. We defined a good response as at least a 50% drop in the ICIQ-LUTS-QoL Part A score six months after BoNT/A injection. We chose a relative change in this score to distinguish between good and poor responders, as, in our opinion, it is the most comprehensive patient-reported outcome measure. Rather than focusing on the severity of specific OAB symptoms, the ICIQ-LUTS-QoL Part A evaluates the overall impact of the disease manifestations on various domains of a patient’s life [35,36]. Hence, a post-treatment decrease in the score is a holistic marker of improvement. We chose at least a 50% decrease in the ICIQ-LUTS-QoL Part A score as a value distinguishing between good and poor responders to BoNT/A treatment, as in absolute terms, it corresponded to a median 19-point drop, a value more than three times higher than the 6-point minimum clinically important difference for the instrument [51]. A similar approach has been used previously by other authors to analyze the effects of various interventions in patients with urological disorders [16,52,53,54,55]. The analysis identified three potential correlates of a good response: a higher BMI, a higher parity, and a lower Qmax at baseline. The latter parameter has already been identified in previous studies as a predictor of a more favorable response to BoNT/A treatment, along with lower filling pressure, larger baseline voided volumes, the presence of urgency urinary incontinence, lower baseline OAB symptom scores, involuntary contractions of the bladder muscle during filling studies, and the lack of concomitant underactivity or bladder outlet obstruction [18,56,57]. However, large clinical studies and reviews generally find that standard pre-injection urodynamic parameters fail to consistently predict therapeutic responses to BoNT/A treatment [58].

In our series, better responses to BoNT/A treatment were more likely in women with a higher BMI and a higher parity, i.e., the two factors known to be associated with more severe detrusor overactivity and a more severe course of OAB [59,60,61]. These findings are in contrast to the results published previously by other authors, according to whom neither parity [58,62] nor BMI [63] was proven to be a predictor of the therapeutic response to intradetrusor injection of BoNT/A in OAB. In our opinion, the discrepancies mentioned above reflect the lack of a unified definition for a successful outcome of BoNT/A treatment. We also note that no multivariate analysis was conducted in the present study, and the number of potential correlates analyzed was limited. Thus, before formulating any firm conclusions regarding the determinants of response to BoNT/A treatment, the hypotheses derived from the results presented herein should be verified through a multivariate analysis using a large panel of potential predictors.

The present study was underpowered, and no formal sample size calculation was carried out. Another potential limitation is the lack of a separate analysis for polymorphic homozygotes (AA genotype) and the insufficient representation of TA heterozygotes in the sample due to their underrepresentation in the general population. Thus, this study should be considered merely exploratory, and its findings should be interpreted with caution. The recruitment of patients with OAB for genetic testing at our center is ongoing, so conducting an analysis involving all three genotypes of the rs12249854 polymorphism in an adequately powered sample is only a matter of time. When analyzing the responses to BoNT/A treatment presented herein, one should consider potential confounders, such as a history of previous pharmacotherapy for OAB, especially with anticholinergic agents, and comorbidities, including the type of underlying neurological condition in the nOAB group. We did not consider these factors in the exploratory analysis, given the small size and considerable heterogeneity of our cohort. Finally, it needs to be stressed that this was a single-center study, and therefore, its results should be generalized with caution.

## 4. Materials and Methods

### 4.1. Ethics

The protocol of the study was approved by the Local Bioethics Committee at Pomeranian Medical University in Szczecin (decision no. KB-1112/77/15; 22 June 2015). All patients gave their written informed consent to participate in the study. All study procedures conformed to the most recent version of the Declaration of Helsinki.

### 4.2. Patients

The study included all consecutive female patients with OAB who qualified for a BoNT/A injection at our department between June 2015 and June 2026. Both patients with iOAB (i.e., urgency accompanied by increased daytime frequency and/or nocturia, with or without urinary incontinence, in the absence of urinary tract infection or other detectable disease) and those with nOAB (i.e., in whom the symptoms mentioned above occurred concomitantly with a clinically relevant neurological disease) were eligible for analysis. Other inclusion criteria of the study were detrusor overactivity confirmed by urodynamic testing and intolerance to or lack of an adequate response to previous pharmacological treatment for OAB. Patients were excluded from the analysis if they had received BoNT/A treatment in the past, presented with mixed urinary incontinence, a urinary tract infection, bladder stones, bladder cancer, or urinary retention, or had a history of previous urogynecological surgery. The list of collected patient characteristics included age, body height, body mass, and body mass index (BMI) (calculated as body mass (kg)/height (m^2^)), as well as number of pregnancies, deliveries, and cesarean sections.

### 4.3. Intradetrusor Injection of BoNT/A

The treatment was carried out in an inpatient setting. All intradetrusor injections of BoNT/A were performed by the same experienced urogynecologist (S.C.). One hundred units of BoNT/A (Botox^®^, Allergan Inc., Irvine, CA, USA) were diluted in 10 mL of normal saline (0.9% NaCl). After placing the patient under general anesthesia, a rigid cystoscope and needle (injeTak^®^, Laborie, Portsmouth, NH, USA) were used to inject BoNT/A into the detrusor muscle at 20 sites (5 units of BoNT/A per site) spaced approximately evenly over the bladder, including the dome but sparing the trigone. Oral fosfomycin prophylaxis (3 g) was administered daily for three consecutive days, beginning the day before the BoNT/A injection.

### 4.4. Pre- and Post-Treatment Evaluation

Before the intradetrusor injection of BoNT/A and six months thereafter, each patient rated frequency, nocturia, urgency, and urge incontinence and completed a survey comprising the ICIQ-OAB questionnaire and the International Consultation on Incontinence Questionnaire–Lower Urinary Tract Symptoms–Quality of Life (ICIQ-LUTS-QoL) questionnaire. In both questionnaires, Part A assessed symptom severity, and Part B assessed cumulative anxiety in the patient. The higher the scores on the questionnaires, the worse the symptom profile (ICIQ-OAB) and/or the greater the negative impact of OAB on quality of life (ICIQ-LUTS-QoL). For the ICIQ-OAB, the total raw scores in Part A ranged from 0 to 16 points, and in Part B, from 0 to 40 points. With ICIQ-LUTS-QoL, the total raw scores in Part A ranged from 0 to 76 points, and in Part B, from 0 to 190 points.

Before the BoNT/A injection and six months thereafter, urodynamic parameters were determined according to ICS standards in all OAB patients. Urodynamic testing was performed by the same investigator (S.C.) after confirming a normal result on urinalysis. Two types of Menfis bioMedica catheters (9 Fr and 12 Fr) were used. One 9 Fr catheter was inserted into the urinary bladder to perform flow cystometry and measure flow pressure. A second 12 Fr catheter was inserted into the rectum to measure intra-abdominal pressure. The urodynamic examination was performed with the patient in the gynecological position. During the examination, 0.9% physiological saline was administered into the urinary bladder at a flow rate of 50–75 mL/min. A cough stress test and Valsalva maneuver were performed after administering the first 50 mL of fluid and repeated after every additional 100 mL. The micturition phase started after maximum filling of the bladder. The patient voided into a container placed on a scale, and the uroflow curve and residual urine volume were determined. Urodynamic parameters, maximum cystometric capacity (MCC), post-void residual (PVR), first sensation of bladder filling (FSBF), maximum flow rate (Qmax), average flow rate (Qave), detrusor pressure at maximum flow during the voiding phase (Pdet at Qmax), and maximum detrusor pressure during the filling phase (Pdet-max) were determined using the Libra + system (Medical Measurement Systems B.V., Amsterdam, The Netherlands).

### 4.5. Genotyping

Before the injection of BoNT/A, a buccal swab was obtained from each patient. Genomic DNA was extracted from buccal epithelial cells using a commercially available DNA isolation kit (PrepFiler^®^ Express Forensic DNA Extraction Kit, Applied Biosystems, Life Technologies Polska, Warsaw, Poland) according to the manufacturer’s instructions. The single nucleotide polymorphism (rs12249854) in the *HECTD2* gene was identified using real-time PCR with the C_30760887_10 TaqMan^®^ SNP genotyping assay (TaqMan^®^ Pre-Designed SNP Genotyping Assay, Thermo Fisher Scientific, Waltham, MA, USA). Amplification was carried out on 96-well plates using a QuantStudio^TM^ 3 Real-Time PCR system (Applied Biosystems, Life Technologies Polska, Warsaw, Poland). The reaction mix, with a final volume of 10 μL, consisted of 5 μL of ProAmp Master Mix (Applied Biosystems, Life Technologies Polska, Warsaw, Poland), 0.25 μL of TaqMan SNP Assay (Applied Biosystems, Life Technologies Polska, Warsaw, Poland), 3.75 μL of nuclease-free water, and 1 μL of genomic DNA. The temperature profile was as follows: 30 s at 60 °C, 10 min at 95 °C, 15 s at 92 °C, and 1 min at 60 °C. A total of 40 cycles were conducted before a final measurement of fluorescence was taken. The genotypes were identified automatically with QuantStudio^TM^ Design & Analysis Software v1.5.3, based on the normalized intensity of FAM dye plotted against the normalized intensity of VIC dye. The identity of each genotype was verified by two independent researchers.

### 4.6. Statistical Analyses

There was no formal sample size calculation in this study. The normality of quantitative variable distributions was assessed using the Shapiro–Wilk test. Statistical characteristics of quantitative variables are presented as medians and lower and upper quartiles. As the vast majority of the quantitative variables were not normally distributed, the Mann–Whitney U-test was used for between-group comparisons. The effect of the *HECTD2* genotype on quantitative outcome measures was tested using linear regression analysis, with OAB type as a cofactor. The distributions of qualitative variables were compared using the chi-squared test or Fisher’s exact test. The main effects of BoNT/A treatment, OAB type, and genotype, and their interactions on the outcome variables (symptom scores, questionnaire scores, and urodynamic parameters) were analyzed using repeated-measures analysis of variance (ANOVA). No correction for multiple comparisons was applied; all *p*-values are unadjusted; and the analyses are exploratory and hypothesis-generating. All calculations were carried out with Stata SE 18.0 (StataCorp, College Station, TX, USA), with the threshold of statistical significance for all tests set at *p* < 0.05.

## 5. Conclusions

The rs12249854 polymorphism in the *HECTD2* gene does not appear to be a correlate of response to BoNTA treatment. However, given the very small sample size and the underrepresentation of the minor rs12249854 allele (A allele), the study should be considered merely exploratory, and its findings should be interpreted with caution. Compared with individuals with iOAB, patients with nOAB seem to have a worse pretreatment urodynamic profile in terms of Pdet and Qmax. Based on a 6-month observation, these differences between OAB types might be at least partially offset by BoNT/A treatment. Individuals with a lower baseline Qmax, a higher BMI, and a higher parity may respond to BoNT/A treatment better than other patients, but this hypothesis needs to be verified through a multivariate analysis in an adequately powered study, optimally a multicenter one, including a large panel of potential predictors. Future studies should also verify the role of the rs12249854 polymorphism in a larger group of OAB patients, including those with the AA genotype. Finally, efforts should focus on developing an accurate prediction model for OAB botulinum treatment.

## Figures and Tables

**Table 1:** Basic characteristics of female OAB patients, stratified by *HECTD2*:rs12249854 genotype.

Variable	All Patients (*n* = 156)	*HECTD2*:rs12249854 Genotype		*p*
		TT	TA	
		(*n* = 137)	(*n* = 19)	
Age [years]	59.5 (49.5:66.5)	59 (49:66)	63 (51:70.5)	0.230
BMI [kg/m^2^]	26.9 (24.2:30.5)	26.9 (24.2:30.8)	27.2 (25.3:29.1)	0.976
Pregnancies, *n*	2 (1:3)	2 (1:3)	2 (2:3)	0.304
Deliveries, *n*	2 (1:2)	2 (1:2)	2 (2:2)	0.333
Cesarean sections, *n*	0 (0:0)	0 (0:0)	0 (0:0)	0.980

BMI, body mass index. Data presented as medians (lower quartile:upper quartile). The “*p*” values are for Mann–Whitney tests with a null hypothesis of no difference between *HECTD2* wild-type homozygotes (TT) and TA heterozygous females. No correction for multiple comparisons was applied; all *p*-values are unadjusted; and the analyses are exploratory and hypothesis-generating.

**Table 2:** ICIQ-OAB and ICIQ-LUTS-QoL questionnaire scores before and six months after intradetrusor injection of botulinum neurotoxin type A (BoNT/A) in female OAB patients, stratified by *HECTD2*:rs12249854 genotype.

Variable	Time Code	All Patients (*n* = 156)	*HECTD2*:rs12249854 Genotype		*p*
			TT (*n* = 137)	TA (*n* = 19)	
ICIQ-OAB, Part A (range: 0 to 16)	0	11 (10:13)	11 (10:13)	13 (11:14)	0.010
	6	5.5 (4:8)	5 (3:8)	6 (5:7)	0.216
	∆_6–0_	−6 (−4:−7)	−5 (−4:−7)	−6 (−3:−9)	0.515
	%∆_6–0_	−50 (−30:−66.7)	−50 (−30:−66.7)	−50 (−28.6:−63.6)	0.705
ICIQ-OAB, Part B (range: 0 to 40)	0	29 (25:31)	29 (25:31)	31 (29:31)	0.092
	6	13.5 (5:24)	13 (5:24)	15 (10:21)	0.485
	∆_6–0_	−12.5 (−2.5:−21)	−12 (−3:−22)	−16 (−2:−19)	0.913
	%∆_6–0_	−50 (−12.5:−80)	−50 (−12.5:−82.3)	−51.6 (−6.2:−65.5)	0.732
ICIQ-LUTS-QoL, Part A (range: 0 to 76)	0	57 (47:65)	56 (46:65)	60 (57:72)	0.024
	6	35 (24.5:49)	35 (23:49)	35 (32:49)	0.374
	∆_6–0_	−20.5 (−6:−30)	−20 (−6:−29)	−26 (−4:−31)	0.444
	%∆_6–0_	−38.5 (−10.3:−55.3)	−38.2 (−10.3:−55.6)	−43.1 (−11.1:−50)	0.727
ICIQ-LUTS-QoL, Part B (range: 0 to 190)	0	139.5 (104:165)	138 (100:164)	150 (122:185)	0.116
	6	38 (8:110)	38 (7:110)	70 (16:91)	0.566
	∆_6–0_	−85 (−23.5:−123.5)	−85 (−23:−123)	−100 (−24:−127)	0.633
	%∆_6–0_	−62.6 (−20:−93)	−65.7 (−18.7:−94.1)	−58.8 (−20.9:−89.3)	0.845

Time codes: 0 = before injection; 6 = 6 months after injection; ∆_6–0_ = absolute difference (%∆_6–0_ = relative difference), defined as the value at 6 months after injection minus the value before injection. ICIQ-OAB, International Consultation on Incontinence Questionnaire–Overactive Bladder; ICIQ-LUTS-QoL, International Consultation on Incontinence Questionnaire–Lower Urinary Tract Symptoms–Quality of Life; BoNT/A, botulinum neurotoxin type A; OAB, overactive bladder; *HECTD2*, gene encoding HECT domain E3 ubiquitin ligase. Data presented as medians (lower quartile:upper quartile). The “*p*” values are for OAB type-adjusted regression analyses with a null hypothesis of no difference between *HECTD2* wild-type homozygotes (TT) and TA heterozygous females. No correction for multiple comparisons was applied; all *p*-values are unadjusted; and the analyses are exploratory and hypothesis-generating.

**Table 3:** Urodynamic assessment before and six months after intradetrusor injection of botulinum neurotoxin type A (BoNT/A) in female OAB patients, stratified by *HECTD2*:rs12249854 genotype.

Variable	Time Code	All Patients (*n* = 156)	*HECTD2*:rs12249854 Genotype		*p*
			TT (*n* = 137)	TA (*n* = 19)	
MCC [mL]	0	215 (166:244)	208 (157:244)	234 (198:254)	0.058
	6	349.5 (312:367)	345 (312:367)	356 (312:367)	0.749
	∆_6–0_	130.5 (93.5:178)	135 (94:191)	114 (78:140)	0.175
	%∆_6–0_	37.1 (27.6:52.8)	37.6 (27.7:54.5)	34.3 (25:39.3)	0.139
FSBF [mL]	0	56 (28:98)	54 (28:95)	60 (38:138)	0.100
	6	145 (134:157)	156 (134:157)	137 (134:156)	0.345
	∆_6–0_	86.5 (35:116.5)	95 (37:118)	58 (20:93)	0.054
	%∆_6–0_	62.7 (28.4:79.5)	64.2 (30.9:80.1)	40.8 (11.2:68.5)	0.129
Qmax [mL/s]	0	17 (16:18)	17 (16:19)	17 (16:18)	0.438
	6	14 (13:17)	14 (13:17)	14 (13:18)	0.749
	∆_6–0_	−2 (−1:−5)	−2 (−1:−5)	−3 (2:−5)	0.411
	%∆_6–0_	−15.4 (−5.6:−35.7)	−15.4 (−5.6:−35.7)	−20 (11.1:−33.3)	0.467
Qave [mL/s]	0	8 (8:9)	8 (8:9)	8 (8:9)	0.501
	6	8 (8:11)	8 (8:11)	8 (8:11)	0.527
	∆_6–0_	0 (−1:1)	0 (−1:1)	0 (−2:3)	0.998
	%∆_6–0_	0 (−12.5:11.8)	0 (−12.5:11.1)	0 (−33.3:27.3)	0.508
Pdet at Qmax [cm H_2_O]	0	37 (35:46)	37 (34:46)	37 (35:53)	0.688
	6	20 (19:21)	20 (19:21)	19 (19:21)	0.814
	∆_6–0_	−17 (−15:−25.5)	−17 (−15:−25)	−18 (−13:−34)	0.643
	%∆_6–0_	−89.5 (−72.6:−130)	−88.9 (−73.7:−130)	−94.7 (−68.4:−178.9)	0.615
Pdet-max [cm H_2_O]	0	51 (37:68)	50 (37:68)	52 (42:72)	0.597
	6	27 (26:28)	27 (26:28)	27 (27:28)	0.553
	∆_6–0_	−23 (−10:−40.5)	−22 (−10:−40)	−24 (−15:−44)	0.468
	%∆_6–0_	−82.7 (−37.3:−141)	−83.3 (−37:−133.3)	−82.1 (−55.6:−157.1)	0.354

Time codes: 0 = before injection; 6 = 6 months after injection; ∆_6–0_ = absolute difference (%∆_6–0_ = relative difference), defined as the value at 6 months after injection minus the value before injection. BoNT/A, botulinum neurotoxin type A; OAB, overactive bladder; *HECTD2*, gene encoding HECT domain E3 ubiquitin ligase; MCC, maximum cystometric capacity; FSBF, first sensation of bladder filling; Qmax, maximum flow rate; Qave, average flow rate; Pdet at Qmax, detrusor pressure at maximum flow; Pdet-max, maximum detrusor pressure. Data presented as medians (lower quartile:upper quartile). The “*p*” values are for OAB type-adjusted regression analyses with a null hypothesis of no difference between *HECTD2* wild-type homozygotes (TT) and TA heterozygous females. No correction for multiple comparisons was applied; all *p*-values are unadjusted; and the analyses are exploratory and hypothesis-generating.

**Table 4:** Basic characteristics of female patients with idiopathic (iOAB) and neurogenic (nOAB) overactive bladder syndrome.

Variable	iOAB	nOAB	*p*
	(*n* = 96)	(*n* = 60)	
Age [years]	60 (51:67)	59 (46:65.5)	0.118
BMI [kg/m^2^]	27.1 (24.6:30.5)	26.5 (23.9:30.3)	0.256
Pregnancies, *n*	2 (2:3)	2 (1:3)	0.508
Deliveries, *n*	2 (1:2)	2 (1:2)	0.729
Cesarean sections, *n*	0 (0:0)	0 (0:0)	0.447

iOAB, idiopathic overactive bladder; nOAB, neurogenic overactive bladder; BMI, body mass index. Data presented as medians (lower quartile:upper quartile). The “*p*” values are for Mann–Whitney tests with a null hypothesis of no difference between iOAB and nOAB. No correction for multiple comparisons was applied; all *p*-values are unadjusted; and the analyses are exploratory and hypothesis-generating.

**Table 5:** ICIQ-OAB and ICIQ-LUTS-QoL questionnaire scores before and six months after intradetrusor injection of botulinum neurotoxin type A (BoNT/A) in female patients with idiopathic (iOAB) and neurogenic (nOAB) overactive bladder syndrome.

Variable	Time Code	iOAB	nOAB	*p*
		(*n* = 96)	(*n* = 60)	
ICIQ-OAB, Part A	0	11 (10:13)	12 (10:13.5)	0.366
(range: 0 to 16)	6	5 (4:7)	6 (3:9)	0.413
	∆_6–0_	−6 (−4:−7)	−5 (−3:−8)	0.644
	%∆_6–0_	−50 (−32:−64.3)	−45.8 (−27.6:−66.7)	0.655
ICIQ-OAB, Part B	0	29 (25:31)	29 (25:31)	0.875
(range: 0 to 40)	6	13 (5:22)	15 (6:28.5)	0.087
	∆_6–0_	−13.5 (−4:−22.5)	−9 (−1:−19)	0.067
	%∆_6–0_	−51.6 (−20.2:−83.3)	−35.4 (−4.4:−72.1)	0.077
ICIQ-LUTS-QoL, Part A	0	57 (46.5:65)	56.5 (47–65)	0.871
(range: 0 to 76)	6	33.5 (23.5:47)	38 (26:51.5)	0.416
	∆_6–0_	−25 (−6:−30)	−17.5 (−5.5:−26)	0.306
	%∆_6–0_	−39.3 (−10.4:−55.6)	−31.8 (−8.3:−52.9)	0.424
ICIQ-LUTS-QoL, Part B	0	139.5 (111:165)	139.5 (98.5:165)	0.824
(range: 0 to 190)	6	32 (8:100.5)	59 (8.5:126.5)	0.252
	∆_6–0_	−91 (−28:−127)	−64.5 (−18.5:−110)	0.186
	%∆_6–0_	−71.4 (−24.2:−93.3)	−56.7 (−13.3:−92.6)	0.321

Time codes: 0 = before injection; 6 = 6 months after injection; ∆_6–0_ = absolute difference (%∆_6–0_ = relative difference), defined as the value at 6 months after injection minus the value before injection. ICIQ-OAB, International Consultation on Incontinence Questionnaire–Overactive Bladder; ICIQ-LUTS-QoL, International Consultation on Incontinence Questionnaire–Lower Urinary Tract Symptoms–Quality of Life; BoNT/A, botulinum neurotoxin type A; iOAB, idiopathic overactive bladder; nOAB, neurogenic overactive bladder. Data presented as medians (lower quartile:upper quartile). The “*p*” values are for Mann–Whitney tests with a null hypothesis of no difference between iOAB and nOAB. No correction for multiple comparisons was applied; all *p*-values are unadjusted; and the analyses are exploratory and hypothesis-generating.

**Table 6:** Urodynamic assessment before and six months after intradetrusor injection of botulinum neurotoxin type A (BoNT/A) in female patients with idiopathic (iOAB) and neurogenic (nOAB) overactive bladder syndrome.

Variable	Time Code	iOAB	nOAB	*p*
		(*n* = 96)	(*n* = 60)	
MCC [mL]	0	216.5 (168:248)	209.5 (160:240)	0.412
	6	356 (344:367)	345 (301:367)	0.271
	∆_6–0_	130.5 (94:184.5)	131 (86.5:177.5)	0.728
	%∆_6–0_	37.8 (27.8:54.2)	36.7 (27.5:51.3)	0.904
FSBF [mL]	0	53 (28:98.5)	58 (34:96.5)	0.520
	6	137 (134:157)	156 (134:157)	0.486
	∆_6–0_	92 (36:117)	80.5 (35:114)	0.793
	%∆_6–0_	64.2 (27.7:80.9)	60 (28.4:74.2)	0.615
Qmax [mL/s]	0	18 (15:19)	17 (16:18)	0.257
	6	14 (13:17)	14 (13:17)	0.079
	∆_6–0_	−2.5 (−0.5:−5)	−2 (−1:−4)	0.846
	%∆_6–0_	−16.5 (−2.8:−38.5)	−15.4 (−5.7:−32)	0.967
Qave [mL/s]	0	8 (8:9)	8 (8:9)	0.922
	6	8 (8:11)	8 (8:9)	0.067
	∆_6–0_	0 (−1:2)	0 (−1:1)	0.297
	%∆_6–0_	0 (−12.5:18.2)	0 (−12.5:11.1)	0.276
Pdet at Qmax [cm H_2_O]	0	36 (34:43)	38.5 (36:46.5)	0.013
	6	20 (19:20)	20 (19:21)	0.186
	∆_6–0_	−16.5 (−14:−24)	−18 (−15.5:−26.5)	0.112
	%∆_6–0_	−85 (−71.4:−122.5)	−92.4 (−75:−130.8)	0.277
Pdet-max [cm H_2_O]	0	48 (37:66)	56 (41:79.5)	0.095
	6	27 (26:28)	27 (26:34)	0.209
	∆_6–0_	−22 (−9.5:−39.5)	−25.5 (−12:−44.5)	0.220
	%∆_6–0_	−80 (−36.4:−121.9)	−92.6 (−41.2:−147.9)	0.352

Time codes: 0 = before injection; 6 = 6 months after injection; ∆_6–0_ = absolute difference (%∆_6–0_ = relative difference), defined as the value at 6 months after injection minus the value before injection. BoNT/A, botulinum neurotoxin type A; iOAB, idiopathic overactive bladder; nOAB, neurogenic overactive bladder; MCC, maximum cystometric capacity; FSBF, first sensation of bladder filling; Qmax, maximum flow rate; Qave, average flow rate; Pdet at Qmax, detrusor pressure at maximum flow; Pdet-max, maximum detrusor pressure. Data presented as medians (lower quartile:upper quartile). The “*p*” values are for Mann–Whitney tests with a null hypothesis of no difference between iOAB and nOAB. No correction for multiple comparisons was applied; all *p*-values are unadjusted; and the analyses are exploratory and hypothesis-generating.

**Table 7:** Characteristics and urodynamic assessment of female OAB patients, stratified by response to a single intradetrusor injection of botulinum neurotoxin type A (BoNT/A).

Variable	Response to BoNT/A		*p*
	Good (*n* = 50)	Poor (*n* = 106)	
Age [years]	63 (55:66)	58 (49:67)	0.063
BMI [kg/m^2^]	28.1 (25.9:32.4)	26.1 (24.1:29.7)	0.008
Pregnancies, *n*	2.5 (2:3)	2 (1:3)	0.004
Deliveries, *n*	2 (2:3)	2 (1:2)	0.006
Cesarean sections, *n*	0 (0:0)	0 (0:0)	0.393
iOAB, *n* (%)	32 (64.0%)	64 (60.4%)	0.664
*HECTD2* wild-type genotype, *n* (%)	45 (90.0%)	92 (86.8%)	0.794
MCC _0_ [mL]	204 (147:240)	222 (170:248)	0.114
MCC _6_ [mL]	345 (343:367)	356 (312:367)	0.550
MCC ∆_0–6_ [mL]	141.5 (94:207)	127 (93:177)	0.154
MCC %∆_0–6_ [%]	40.4 (27.2:57.9)	36.2 (28.1:50.7)	0.166
FSBF _0_ [mL]	47 (22:92)	56.5 (34:99)	0.085
FSBF _6_ [mL]	156 (134:157)	137 (134:157)	0.558
FSBF ∆_6–0_ [mL]	102.5 (55:122)	78 (35:109)	0.039
FSBF %∆_6–0_ [%]	66.5 (37.2:85.9)	59.1 (23.6:74.6)	0.054
Qmax _0_ [mL/s]	17 (15:18)	18 (16:19)	0.026
Qmax _6_ [mL/s]	14 (13:15)	15 (13:17)	0.012
Qmax ∆_6–0_ [mL/s]	−2 (−1:−4)	−2 (0:−5)	0.808
Qmax %∆_0–6_ [%]	−15.4 (−6.2:−33.3)	−15.4 (0:−38.5)	0.646
Qave _0_ [mL/s]	8 (8:9)	8 (8:9)	0.793
Qave _6_ [mL/s]	8 (8:9)	8 (8:11)	0.014
Qave ∆_6–0_ [mL/s]	0 (−1:1)	0 (−1:2)	0.087
Qave %∆_6–0_ [%]	0 (−12.5:10)	0 (−12.5:18.2)	0.077
Pdet at Qmax _0_ [cm H_2_O]	36 (34:46)	37 (35:45)	0.509
Pdet at Qmax _6_ [cm H_2_O]	20 (19:20)	20 (19:21)	0.837
Pdet at Qmax ∆_6–0_ [cm H_2_O]	−17 (−15:−25)	−17.5 (−15:−26)	0.647
Pdet at Qmax ∆_6–0_ [%]	−83.7 (−75:−119)	−89.5 (−71.4:−130)	0.784
Pdet-max _0_ [cm H_2_O]	51 (36:75)	50 (37:67)	0.959
Pdet-max _6_ [cm H_2_O]	27 (26:29)	27 (26:28)	0.587
Pdet-max ∆_6–0_ [cm H_2_O]	−24.5 (−9:−43)	−22 (−10:−38)	0.720
Pdet-max %∆_6–0_ [%]	−94.2 (−35.7:−150)	−76.8 (−38.5:−133.3)	0.630

Time codes: 0 = before injection; 6 = 6 months after injection; ∆_6–0_ = absolute difference (%∆_6–0_ = relative difference), defined as the value at 6 months after injection minus the value before injection. OAB, overactive bladder; BoNT/A, botulinum neurotoxin type A; BMI, body mass index; iOAB, idiopathic overactive bladder; *HECTD2*, gene encoding HECT domain E3 ubiquitin ligase; MCC, maximum cystometric capacity; FSBF, first sensation of bladder filling; Qmax, maximum flow rate; Qave, average flow rate; Pdet at Qmax, detrusor pressure at maximum flow; Pdet-max, maximum detrusor pressure. Data presented as medians (lower quartile:upper quartile). The “*p*” values are for Mann–Whitney tests or chi-squared/Fisher’s exact test with a null hypothesis of no difference between good and poor response to botulinum neurotoxin type A. Good response: relative decrease between ICIQ-LUTS-QoL, Part A scores at 6 months after injection and before injection (%∆_6–0_) of at least 50%; poor response: relative decrease between ICIQ-LUTS-QoL, Part A scores at 6 months after injection and before injection (%∆_6–0_) of less than 50%. No correction for multiple comparisons was applied; all *p*-values are unadjusted; and the analyses are exploratory and hypothesis-generating.

## Data Availability

The data are available from the corresponding author upon reasonable request.

## Supplementary Materials

The following supporting information can be downloaded at: https://www.mdpi.com/article/10.3390/ijms27219571/s1.

## Abbreviations

The following abbreviations are used in this manuscript:

ICS	International Continence Society
OAB	Overactive bladder
iOAB	Idiopathic overactive bladder
nOAB	Neurogenic overactive bladder
BoNT/A	Botulinum neurotoxin type A
LC	Light chain
HC	Heavy chain
SV2	Synaptic vesicle protein 2
SNAP-25	Synaptosomal-associated 25 kDa protein
VCIP135	Valosin-containing protein complex-interacting protein 135 kDa
HECT	Homologous to E6-AP carboxy-terminus
BMI	Body mass index
ICIQ-OAB	International Consultation on Incontinence Questionnaire–Overactive Bladder
ICIQ-LUTS-QoL	International Consultation on Incontinence Questionnaire–Lower Urinary Tract Symptoms–Quality of Life
MCC	Maximum cystometric capacity
PVR	Post-void residual
FSBF	First sensation of bladder filling
Qmax	Maximum flow rate
Qave	Average flow rate
Pdet at Qmax	Detrusor pressure at maximum flow
Pdet-max	Maximum detrusor pressure
ANOVA	Analysis of variance
