# Supplementary material for: Effect of the rs12249854 Polymorphism in the HECTD2 Gene and Selected Clinical Variables on the Response to Intradetrusor Injection of Botulinum Neurotoxin Type a in Female Patients with Overactive Bladder: A Single-Center Prospective Study
Source: Int J Mol Sci. 2026 Oct 26;27(21):9571. doi: 10.3390/ijms27219571 (PMC13763376; doi:10.3390/ijms27219571)
Supplement: Supplementary file 1 [file ijms-27-09571-s001.zip › ijms-4566940-supplementary.pdf]

Table S1. Main effects of botulinum neurotoxin type A injection, *HECTD2*:rs12249854 polymorphism, and overactive bladder type, and the effects of interactions between these variables on a change in patient-reported outcome measures within 6 months from a single intra-detrusor injection of botulinum neurotoxin type A.

**Outcome variable**	**BoNT/A**	rs12249854	OAB	BoNT/A x rs12249854	BoNT/A x OAB	rs12249854 x OAB	BoNT/A x rs12249854 x OAB
ICIQ-OAB part A	F = 178.49	F = 4.19	F = 1.32	F = 0.49	F = 0.00	F = 0.03	F = 0.07
	p < 0.001	p = 0.042	p = 0.252	p = 0.485	p = 0.973	p = 0.865	p = 0.792
ICIQ-OAB part B	F = 72.52	F = 1.03	F = 0.93	F = 0.32	F = 0.08	F = 0.02	F = 1.08
	p < 0.001	p = 0.311	p = 0.336	p = 0.575	p = 0.782	p = 0.885	p = 0.301
ICIQ-LUTS-QoL part A	F = 64.91	F = 5.37	F = 2.53	F = 0.40	F = 0.12	F = 1.56	F = 0.02
	p < 0.001	p = 0.022	p = 0.114	p = 0.530	p = 0.729	p = 0.214	p = 0.901
ICIQ-LUTS-QoL part B	F = 67.33	F = 2.27	F = 1.45	F = 0.06	F = 0.90	F = 0.55	F = 0.17
	p < 0.001	p = 0.134	p = 0.230	p = 0.810	p = 0.345	p = 0.458	p = 0.678

ICIQ-OAB, International Consultation on Incontinence Questionnaire-Overactive Bladder; ICIQ-LUTS-QoL, International Consultation Incontinence Questionnaire-Lower Urinary Tract Symptoms-Quality of Life; BoNT/A, botulinum neurotoxin type A; rs12249854, *HECTD2*:rs12249854 polymorphism (wild-type allele homozygote TT, polymorphic heterozygote TA); OAB, type of overactive bladder (iOAB, nOAB). The "F" and "p" values determined on repeated-measures analysis of variance (ANOVA).

Table S2. Main effects of botulinum neurotoxin type A injection, *HECTD2*:rs12249854 polymorphism, and overactive bladder type, and the effects of interactions between these variables on a change in patient-reported outcome measures within 6 months from a single intra-detrusor injection of botulinum neurotoxin type A.

**Outcome variable**	**BoNT/A**	rs12249854	OAB	BoNT/A x rs12249854	BoNT/A x OAB	rs12249854 x OAB	BoNT/A x rs12249854 x OAB
MCC	F = 191.67	F = 3.93	F = 0.01	F = 1.37	F = 0.03	F = 1.51	F = 0.01
	p < 0.001	p = 0.059	p = 0.910	p = 0.244	p = 0.872	p = 0.221	p = 0.916
FSBF	F = 67.75	F = 1.51	F = 1.00	F = 1.27	F = 1.86	F = 0.76	F = 2.84
	p < 0.001	p = 0.222	p = 0.318	p = 0.134	p = 0.175	p = 0.386	p = 0.094
Qmax	F = 14.02	F = 0.55	F = 3.47	F = 0.26	F = 0.04	F = 0.22	F = 0.26
	p < 0.001	p = 0.461	p = 0.065	p = 0.612	p = 0.844	p = 0.636	p = 0.612
Qave	F = 0.00	F = 0.68	F = 1.66	F = 0.00	F = 0.35	F = 0.00	F = 0.00
	p = 0.960	p = 0.411	p = 0.199	p = 0.978	p = 0.552	p = 0.956	p = 0.954
Pdet at Qmax	F = 217.85	F = 0.02	F = 0.23	F = 0.03	F = 0.06	F = 0.97	F = 0.31
	p < 0.001	p = 0.881	p = 0.632	p = 0.867	p = 0.810	p = 0.327	p = 0.579
Pdet-max	F = 97.52	F = 0.00	F = 0.93	F = 0.17	F = 0.18	F = 0.36	F = 0.28
	p < 0.001	p = 0.966	p = 0.335	p = 0.681	p = 0.669	p = 0.549	p = 0.594

MCC, Maximum Cystometric Capacity; FSBF, First Sensation of Bladder Filling; Qmax, Maximum Flow Rate; Qave, Average Flow Rate; Pdet at Qmax, Detrusor Pressure at Maximum Flow; Pdet-max, Maximum Detrusor Pressure; BoNT/A, botulinum neurotoxin type A; rs12249854, *HECTD2*:rs12249854 polymorphism (wild-type allele homozygote TT, polymorphic heterozygote TA); OAB, type of overactive bladder (iOAB, nOAB). The "F" and "p" values determined on repeated-measures analysis of variance (ANOVA).
